# The Central Carbon and Energy Metabolism of Marine Diatoms

**DOI:** 10.3390/metabo3020325

**Published:** 2013-05-07

**Authors:** Toshihiro Obata, Alisdair R. Fernie, Adriano Nunes-Nesi

**Affiliations:** 1 Max-Planck-Institut für Molekulare Pflanzenphysiologie, Am Mühlenberg 1, Potsdam-Golm 14476, Germany; E-Mail: Fernie@mpimp-golm.mpg.de; 2 Max-Planck Partner Group, Departamento de Biologia Vegetal, Universidade Federal de Viçosa, Viçosa 36570-000, Minas Gerais , Brazil; E-Mail: nunesnesi@ufv.br

**Keywords:** diatoms, *Phaeodactylum tricornutum*, *Thalassiosira pseudonana*, central carbon metabolism, photosynthesis, biofuel, lipid biosynthesis

## Abstract

Diatoms are heterokont algae derived from a secondary symbiotic event in which a eukaryotic host cell acquired an eukaryotic red alga as plastid. The multiple endosymbiosis and horizontal gene transfer processes provide diatoms unusual opportunities for gene mixing to establish distinctive biosynthetic pathways and metabolic control structures. Diatoms are also known to have significant impact on global ecosystems as one of the most dominant phytoplankton species in the contemporary ocean. As such their metabolism and growth regulating factors have been of particular interest for many years. The publication of the genomic sequences of two independent species of diatoms and the advent of an enhanced experimental toolbox for molecular biological investigations have afforded far greater opportunities than were previously apparent for these species and re-invigorated studies regarding the central carbon metabolism of diatoms. In this review we discuss distinctive features of the central carbon metabolism of diatoms and its response to forthcoming environmental changes and recent advances facilitating the possibility of industrial use of diatoms for oil production. Although the operation and importance of several key pathways of diatom metabolism have already been demonstrated and determined, we will also highlight other potentially important pathways wherein this has yet to be achieved.

## 1. Introduction

Diatoms are unicellular, photosynthetic, eukaryotic algae, which are ubiquitous in the world's oceans and freshwater systems. There are more than 200 genera of living diatoms, and it is estimated that there are approximately 100,000 extant species [1]. Most live pelagically in open water, although some live at the water-sediment interface (benthic) as surface films or even under damp atmospheric conditions. Diatoms are usually microscopic, however, some species including a giant diatom, *Ethmodiscus* spp., can reach up to 1.9 mm in length [2]. Their plastids are surrounded by four membranes and show a gold-brown color due to a high amount of the carotenoid fucoxanthin [3]. Diatoms are characterized by their intricately structured cell walls made of silica with species specific and highly differentiated nano- and micropatterns [4]. Due to the rigidity and architecture of the cell wall, most diatoms are non-motile [4]. As their relatively dense cell walls cause them to readily sink, planktonic forms in open water usually rely on turbulent mixing of the upper layers by the wind to keep them suspended in sunlit surface waters [5]. An active regulation of cellular buoyancy with intracellular lipids is suggested in some species to counter sinking [6].

Taken together the diatoms constitute the most abundant group of marine eukaryotic phytoplankton. They contribute up to 40% of organic matter production in the ocean, which is an equivalent biomass to that of all tropical rain forests on earth [7]. As such, diatoms play important roles in the marine ecosystem, as a primary producer in the marine ecosystem, as a major carbon carrier to the deep ocean to be one of the major components of the so-called biological carbon pump [8], and additionally as a major source of petroleum deposits [9].

Evolutionarily diatoms were derived by a secondary endosymbiotic event, which occurred between an unknown eukaryotic cell and a red alga. In this process the diatom acquired genes from two eukaryotic genomes. In addition there is an increasing body of evidence, which suggests the occurrence of a considerable level of horizontal gene transfer among marine organisms [10,11]. Such an evolutionary background renders algae as a "melting pot" of genes and provides them with a particular opportunity to evolve new gene and metabolic networks [12]. Despite this fact, a large number of diatom proteins are shared by many groups of photosynthetic eukaryotes, namely red alga, green plant and cyanobacterium, and many of them are likely involved in chloroplast functions. The landmark publication of the first diatom genome sequence, that of the centric diatom *Thalassiosira pseudonana* [13] followed by that of the pennate diatom *Phaeodactylum tricornutum* [14], facilitated cross-species and cross-kingdom comparisons at an unprecedented level. Intriguingly, several hundred proteins (806 in *T*. *pseudonana*) can be aligned with mouse proteins but not with proteins of green plants or red alga. These "animal-like" genes were likely derived from the heterotrophic secondary host [13]. In addition, the analysis of *P*. *tricornutum* genome documented the presence of several hundreds of genes considered to be of bacterial origin, more than 300 of which are present also in *T*. *pseudonana*. It is conceivable that diatoms obtained these genes by horizontal gene transfer from bacteria and that this process has been a major driving force during diatom evolution allowing diatoms to take advantage of genes from various sources and to construct a mosaic metabolic network distinct from other photosynthetic organisms including higher plants [14].

Whilst bearing striking similarities in some aspects, the metabolic pathways of diatoms are quite distinct from those of well-studied land plants in many others. Therefore the establishment of biosynthetic pathways and their inherent genetic and metabolic regulatory mechanisms will need to be confirmed for these species. In the following sections we will discuss our current understanding and major knowledge gaps with particular focus on (1) central carbon metabolism, (2) adaptation to environmental conditions and (3) the importance of storage lipids as energy reserves for diatoms and their potential exploitation as a source of biofuel.

## 2. Distinctive Carbon Metabolism in Diatoms

**Figure 1:**
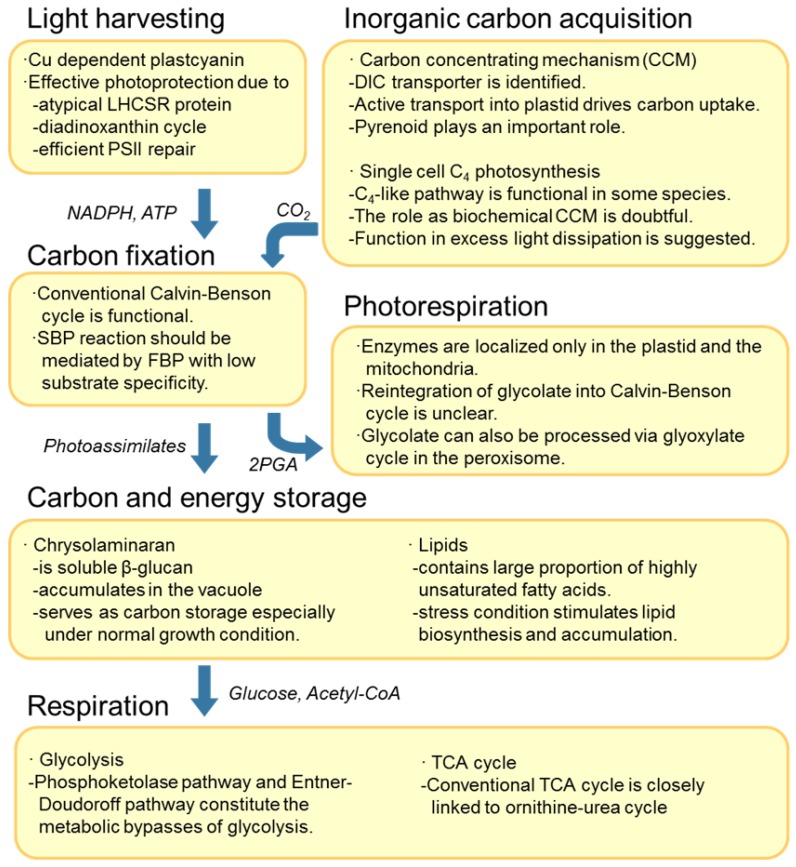
Summary of central carbon and energy metabolism in diatoms. DIC, dissolved inorganic carbon; LHCSR, light harvesting complex stress related; PSII, photosystem II; SBP, sedoheptulose bisphosphatase; FBP, fructose bisphosphatase; 2PGA, 2-phosphoglycerate; TCA, tricarboxylic acid

The genome sequences of two diatoms allow us to estimate the functionality of metabolic pathways and establish a metabolic network model for diatoms. The full set of genes related to carbon metabolism was first suggested on the completion of the *T*. *pseudonana* genome [13] whilst a metabolic model was proposed for *P*. *tricornutum* based on the whole genome sequence [15]. Recent extensions in gene annotation and metabolic pathway reconstruction based on the database of metabolic pathways MetaCyc allowed to fill the gaps apparent in the first model and furthermore led to the elucidation of novel pathways in *P*. *tricornutum* [16], which we detail below. Interrogation of expressed sequence tag and microarray based transcriptome analyses provides first support for the functionality of the pathways [17,18] as do proteomic data for these species [19,20]. These results revealed that whilst some pathways are shared across the lineages of living organisms there are additionally distinctive carbon metabolic pathways in diatoms. In the following section we provide an overview of the general structure of the biosynthetic and core metabolism of diatoms as well as highlighting novelties of the carbon metabolism of these species (Figure 1). Although not directly related to central carbon metabolism we begin by discussing the light harvesting machinery since this is crucial for efficient primary carbon assimilation.

### 2.1. Light Harvesting Machinery to Cope with High Light

The light availability to phytoplanktonic algae is highly variable since they are continuously moved between different depths by the process of vertical mixing. For this reason microalgae must be resilient to short term changes of light intensity especially to the high light which can disrupt photosystem II (PSII) by photoinhibition [21]. Diatoms have been demonstrated to display outstanding capacity to cope with high light. The diadinoxanthin cycle is one of the xanthophyll cycles distributed in the Bacillariophyceae, Xanthophyceae, Haptophyceae and Dinophyceae algal classes and represents the most important photoprotective mechanism of diatoms [22]. The pool size of diadinoxanthin in *P*. *tricornutum* is modulated by the light regime and closely related to non-photochemical quenching under exposure to high light intensity [23]. *P*. *tricornutum* also possesses an atypical light-harvesting complex stress-related (LHCSR) protein which functions in modulation of excess light energy dissipation [24]. Furthermore, diatoms show rapid turnover of multiple PSII subunits including D1 protein and pools of PSII repair cycle intermediates in order to maintain PSII function, especially at the onset of high light exposure [25,26]. This outstanding capacity of diatoms to cope with high light is considered to explain partly the ecological advantage of diatoms and therefore their dominance in the contemporary ocean [27]. However, it is likely that this is an over simplification of the situation and that diatoms have several other features which contribute to their success, some of which may be related to their unique mixture of metabolic pathways.

### 2.2. Inorganic Carbon Acquisition—Biophysical or Biochemical Carbon Concentrating Mechanism?

The diffusion of CO_2_ in water is more than 5,000 times slower than in air and thus greatly limits the carbon acquisition by phytoplankton. The low affinity of RubisCO to CO_2_ and its competing oxygenase reaction further reduce the photoassimilation in aquatic environments even though the RubisCO of diatom shows relatively high affinity for CO_2_ and CO_2_/O_2_ selectivity [28]. The majority of microalgae cope with the limited availability of CO_2_ by developing carbon-concentrating mechanisms (CCMs) to accumulate CO_2_ close to RubisCO [29]. There are two major CCMs proposed. One is a biophysical CCM in which CO_2_ and HCO_3_^−^ are transported as inorganic forms by the aid of transporters and carbonic anhydrases (CAs). Here the enhanced conversion between CO_2_ and HCO_3_^¯^ mediated by CAs and the alkaline stromal pH play crucial roles [30]. The other is a biochemical CCM involving the fixation of HCO_3_^−^ into C_4_ compounds in the manner similar to C_4_ plants [28,31]. Whilst there is cumulative evidence showing the involvement of CCMs in diatoms including active uptake of both CO_2_ and HCO_3_^−^ and single cell C_4_ like metabolism, the underlying mechanisms are still controversial [28,32]. Evidence presented in recent studies and contributing to the debate is presented in the following sections.

#### 2.2.1. Inorganic Carbon Concentrating Mechanism and Ion Pumping

There are many physiological studies indicating active uptake of both HCO_3_^−^ and CO_2_ in diatoms although the preference in the form of inorganic carbon is species specific [31,33,34]. High affinity photosynthesis in *P*. *tricornutum* is severely inhibited by the CA specific inhibitor ethoxyzolamide indicating a crucial role for CA in the CCM of this species [35]. Very recently one of the plasma membrane type solute carrier family proteins in *P*. *tricornutum*, PtSLC4-2 has been shown to stimulate the active uptake of dissolved inorganic carbon and photosynthesis [36]. It is highly conceivable that molecular evidence for biophysical CCM in diatoms will increase in the near future given that the genomes of *P*. *tricornutum* and *T*. *pseudonana* contain numerous genes encoding CAs and putative bicarbonate transporters [13,15]. Reverse genetic methodologies for these species would accelerate the functional analysis of these genes [28,37,38,39]. Biochemical evidence in the form of a ^18^O depletion assay coupled with metabolic modeling indicated that two thirds of net carbon uptake is mediated by the diffusion of CO_2_ and the remaining one third by active HCO_3_^−^ transport. The active transport of carbon into the plastid is predicted specifically as the main driver of the CCM of *P*. *tricornutum* [40]. However, the results of this study also indicate that the efficiency of CCM is not sufficient to achieve half saturation constant of RubisCO in the plastid. The functionality of the pyrenoid, wherein RubisCO is microcompartmented, is suggested to be additionally necessary to fill the gap by achieving efficient use and higher concentration of CO_2_ [40].

#### 2.2.2. Role of C_4_-like Pathway

The unicellular C_4_ photosynthesis in *Thalassiosira* *weissflogii* was proposed based on the rapid ^14^C incorporation into malate and transfer of ^14^C from malate to C_3_ compounds [41]. A set of genes encoding enzymes for C_4_ metabolism were identified in both sequenced diatoms [13,15] and a proteomic study identified phospho*enol*pyruvate carboxykinase (PEPCK) and two phospho*enol*pyruvate carboxylases (PEPCs) as abundant proteins in *T*. *pseudonana* [19]. Although the similar effects of inhibitors for PEPC on these three diatom species suggest the involvement of a C_4_ pathway in carbon fixation [42], the distribution of enzyme activities, regulation of gene expression and results of ^14^C labeling experiments were variable among species and even within the same species [32,43]. Additionally in both sequenced species no enzymes of C_4_ photosynthesis are predicted to localize in plastids [13,15] rendering the function of a C_4_ pathway as an effective biochemical CCM in diatoms equivocal. However, a clearer picture has emerged from a recent study on RNAi line inhibited in the expression of the single gene encoding pyruvate-orthophosphate dikinase (PPDK) in *P. tricornutum* [44]. The photosynthetic affinity for CO_2_ was hardly affected in the RNAi line, suggesting at best a minor contribution of a C_4_ pathway to the CCM of this species. On the other hand the results of this study did suggest that the C_4_ pathway played important roles in the dissipation of excess light energy and in pH homeostasis [44].

### 2.3. The Calvin-Benson Cycle and Photoassimilate Conversion to the Chrysolaminaran Storage Form

Regardless of the participation of a C_4_-like metabolism in inorganic carbon acquisition, CO_2_ must be assimilated by RubisCO and processed via the Calvin-Benson cycle. The diatom genomes encode all conventional enzymes of the Calvin-Benson cycle other than plastidial sedoheptulose bisphosphatase (SBP) [15]. Given that the reaction by SBP can alternatively be mediated by some fructose bisphosphatases with low substrate specificity [15], it seems reasonable to assume that the Calvin-Benson cycle is likely primarily responsible for the carbon fixation of diatoms. The fixed carbon is stored mainly as lipids and as the *β*-1,3 glucan known as chrysolaminaran in these species. Chrysolaminaran is a water soluble polysaccharide which accumulates in the vacuole, in contrast to starch which is crystallized and stored in chloroplast in members of green lineage [45] and floridean starch which accumulates in the cytosol of red algae [46]. Chrysolaminaran represents the primary carbon storage under nutrient replete condition [47], however, as is detailed below, under conditions of stress lipophilic carbon predominates. The biosynthetic pathway of chrysolaminaran has not been fully elucidated yet but a combination of enzyme activity assays and ^14^C labeling experiments suggests that UDP-glucose is the major substrate for the synthesis of this glucan in *Cyclotella cryptica* [48]. Chrysolaminaran accumulates in *Skeletonema costatum* during the day and degrades during the night in concert with elevated glucanase activity [49]. A recent transcriptome analysis revealed the diurnal expression patterns of genes related to storage glucan metabolism in *P*. *tricornutum* [18]. The genes encoding putative UDP-glucose pyrophosphorylase, *β*-1,3 glucan synthase, 1,6-*β*-branching enzyme and enzymes of gluconeogenesis, predicted enzymes for chrysolaminaran catabolism including endo-1,3-*β*-glucosidases reached maximum expression during the dark period [18]. These results indicate transcriptional regulation of enzymes of chrysolaminaran metabolism and furthermore suggest the functionality of the genes predicted to be involved in this pathway. However, direct genetic evidence for these functionalities is currently lacking.

### 2.4. Photorespiration in Two Cellular Compartments

As mentioned above, RubisCO fixes not only CO_2_ but also O_2_ leading to glycolate production. Marine phytoplankton releases a large fraction of glycolate into the water body, resulting in carbon loss. The photorespiratory pathway is required to recycle glycolate back into metabolism. The genes related to the pathway were recently demonstrated to be present and functional in diatoms [50]. The annotation of the enzymes on the basis of their genomic sequence revealed a set of genes for photorespiratory pathway, however, several differences between those in diatoms and in higher plants were observed [15]. The enzymes of the photorespiratory cycle are predicted to be in the plastid and the mitochondria in contrast to higher plants in which these enzymes distribute in four cellular compartments [51]. In diatoms, glycolate can also be metabolized by the glyoxylate cycle located in peroxisomes although the carbon is not integrated into Calvin-Benson cycle. The reintegration should be mediated in a different way in diatoms due to the lack of a gene encoding glycerate kinase which catalyzes the last reaction of the cycle by converting glycerate into 3-P-glycerate [15]. Additionally the fact that diatoms possess a relatively high specificity factor (τ) of RubisCO, a measure of its ability to discriminate CO_2_ from O_2_, suggests a relatively low rate of O_2_ fixation in diatoms [52]. Taken together these observations, the photorespiratory pathway of diatoms, appears to be quite different from that of seed plants both in the reactions involved and extent of operation [53].

### 2.5. Respiration Closely Linked to Other Metabolic Pathways

Glucose produced by degradation of chrysolaminaran is catabolized via glycolysis and the tricarboxylic acid (TCA) cycle to produce ATP and NADH. These pathways are important to maintain the cellular energy metabolism under non-photosynthetic conditions and also to provide a carbon backbone to a number of downstream metabolic pathways. However the studies on these pathways in diatoms are relatively scarce. Recent genome-wide analyses shed light on the respiratory processes and suggested the presence of conventional pathways interrelated with unusual ones.

#### 2.5.1. Presence of Glycolytic Bypasses

Genes encoding a complete cytosolic glycolysis have been identified in *T*. *pseudonana* [13]. In *P*. *tricornutum*, most glycolytic enzymes have been predicted to be localized in both the cytosol and the plastid, while enolase is targeted to the plastid and the mitochondria. This suggests the occurrence of this pathway in plastids, but unlikely in the cytosol [15]. The reactions downstream of glyceraldehyde-3-phosphate (GAP) can also happen in mitochondria [15]. In addition to a conventional glycolytic pathway, recent extension of the metabolic model for diatoms—DiatomCyc—suggested the presence of bypass pathways of glycolysis in these species [16]. One of these bypasses is a phosphoketolase pathway (PKP), which is a catabolic variant of the oxidative pentose phosphate pathway (OPPP). The *P*. *tricornutum* genome encodes a cytosolic xylulose-5-phosphate/fructose-6-phosphate phosphoketokinase (XKP), which potentially catalyze the cleavage of an OPPP intermediate xylulose-5-phosphate to produce acetyl-phosphate and GAP. The XKP gene in *P*. *tricornutum* is homologous to those from cyanobacteria and Lactobacillaceae and no ortholog was found in *T*. *pseudonana*, suggesting the bacterial origin of this gene [16]. However it is important to note that direct experimental evidence of its functionality is still lacking.

Another proposed glycolytic bypass is a mitochondrial Entner-Doudoroff pathway. Following this scheme the 6-phosphogluconate (6PG) produced by the OPPP is dehydrated by 6PG dehydratase and the resulting 2-keto-3-deoxyphosphogluconate (KDPG) is cleaved into pyruvate and GAP by a KDPG aldolase. The functionality of these enzymes has been confirmed by bacterial complementation and enzymatic activity assays and diurnal change of the expression of these genes has also been observed [16,18]. In combination with mitochondrial isoforms of glycolytic enzymes [15], 6PG is also proposed to be degraded to pyruvate within the mitochondria of *P*. *tricornutum* [16].

#### 2.5.2. The TCA Cycle is Intimately Linked to the Ornithine-Urea Cycle

Both sequenced diatoms possess a complete set of genes encoding the mitochondrial TCA cycle [13,15]. All proteins of TCA cycle enzymes, encoded by 14 genes annotated in the genome, were detected in a proteomic analysis in *T*. *pseudonana*, favoring the functionality of the cycle [19]. The expression of TCA cycle related genes showed a coordinated diurnal pattern with the peak at the beginning of dark period, which is in accordance with its function in energy production [18]. Interestingly, the regulation of this pathway has been shown to be associated tightly with the ornithine-urea cycle (OUC). The accumulation of TCA cycle intermediates was closely correlated with that of OUC related metabolites but they were completely uncoupled from OUC endpoints, urea and proline [54]. OUC was therefore proposed to serve as a hub connecting carbon metabolism and nitrogen fixation/remobilization and to play a crucial role to balance the demand for carbon and nitrogen in the natural habitat with fluctuating nitrogen availability [53,54]. In *T*. *pseudonana,* expression of both TCA cycle and OUC related enzymes were up-regulated under nitrogen limitation [20]. Such transcriptional responses of TCA cycle genes were similar to cyanobacteria possessing OUC but distinct from those of *Arabidopsis thaliana* and *Chlamydomonas* *reinhardtii* which lack functional OUC [20]. These results highlighted an important role of the TCA cycle to orchestrate the carbon and nitrogen metabolisms in concert with OUC in diatoms. However, further investigations by reverse genetic approaches are required to assess the relative contributions of the TCA cycle and of the OUC to energy metabolism and to other processes in diatom cells.

## 3. Adaptation of Diatom Carbon Metabolism to a Changing Environment

The environment of phytoplankton affects their growth and metabolism and in turn their effect on the ecosystem [55]. Given that ecological impacts of plankton communities are altered by both their diversity and biogeography [56,57], the growth and metabolic effects of a changing environment on individual algal species are of crucial importance in understanding the interactive influences between algal community and their environment. As stated above, diatoms represent one of the most abundant groups of algae on the basis of both number of individuals and biomass, and therefore are of particular relevance in ecological terms. On the other hand robustness of diatoms against abiotic stresses is likely to be one of the main reasons for their success in the contemporary oceans. In this section we discuss recent studies on the response of diatoms with particular focus on those related to anticipated future environmental changes including increasing atmospheric CO_2_ level and water pollution. The responses of diatoms to high light and macronutrient deficiency are discussed in Section 2.1, Section 2.5, Section 4.1

### 3.1. Elevated CO_2_ Level and Ocean Acidification

The level of atmospheric CO_2_ is rising due to anthropogenic emission of CO_2_ mainly from fossil fuel burning. Under the Intergovernmental Panel on Climate Change (IPCC) CO_2_ emission scenario, atmospheric CO_2_ concentration would reach to 965 ppm coinciding with the decrease of mean surface ocean pH to 7.74 from the preindustrial value of 8.18 [58]. Oceanic photosynthesis plays a crucial role in modulating global warming by fixing atmospheric CO_2_ emitted by anthropogenic activities [7,59]. Rising CO_2_ is considered to enhance aquatic photosynthesis by providing inorganic carbon as substrate [60]. However, the effect is complicated by many factors including the interrelationship between pH and availability of inorganic carbon species, CCM in algae [61] and the fact that rising CO_2_ generally occurs in combination with other environmental changes [62]. Indeed a number of experiments have revealed contradictory results among different growth conditions and diatom species, some demonstrated a stimulative effect of elevated CO_2_ but others showed no change or even negative effects on their growth and photosynthesis (summarized in [61]). Recent experiments investigating long term effects of increased aquatic CO_2_ by decreasing pH to the predicted level at the end of the century were performed in continuous cultures of *T*. *pseudonana* [63]. Only mild effects on the cellular C:N ratio and the expression of a single CA were observed indicating this species to be capable of acclimating to this magnitude of pH change [63]. However, more comprehensive analysis using wider variety of species is required to evaluate the effect of elevated CO_2_ on the growth and primary productivity of diatoms particularly within an ecological context.

### 3.2. Iron Limitation

Primary production in large ocean area especially in the high nutrient low chlorophyll regions is considered to be limited by iron availability. Indeed mesoscale iron fertilization experiments led to phytoplankton blooms mainly consisting of diatoms [64,65,66]. Therefore ocean iron fertilization has been proposed as a method to enhance oceanic carbon fixation to reduce atmospheric CO_2_ and ocean acidification. However, this approach is controversial due to debate on the total balance of CO_2_ fixation and release caused by fertilization. Furthermore environmental risks have been proposed including expanded regions with low oxygen concentration, increased production of N_2_O and possible disruptions of marine ecosystems [67]. It has been experimentally demonstrated that diatom species dominant in coastal region and open oceans employ different strategies to cope with low iron availability. The oceanic diatom *Thalassiosira* *oceanica* permanently employs a photosynthetic apparatus requiring less iron to achieve the equivalent photosynthetic rate [68]. It is achieved by utilizing greater amounts of Cu than coastal diatoms due to the use of Cu containing plastocyanin instead of Fe containing cytochrome *c*_6_ for photosynthesis [69]. On the other hand coastal diatoms respond to iron availability in a transient manner. The coastal diatom *P*. *tricornutum* is known to be highly tolerant to Fe limitation [70]. Non-targeted transcriptomic and metabolomic approaches were applied to explore the biochemical responses in growth-limiting levels of dissolved Fe [71]. This revealed the large scale metabolic rearrangement including the remobilization of carbon and nitrogen from protein and carbohydrate stores and the activation of biosynthesis pathways for antioxidants. The Fe uptake was also activated by increasing the level of the strong Fe chelating glucose oxidation derivative gluconate as well as the up-regulation of iron transporting siderophores [71].

### 3.3. Chemical Pollution

Water pollution arising from industrial and agriculture waste is becoming a major problem in coastal environments, especially in densely populated areas, which are subject to an intensive exposure to human and industrial activity. Recently transcriptomic technologies have been applied in ecotoxicology studies in order to explore the impact of toxic chemicals and to develop the diagnostic markers for accessing ecosystem quality [72]. Diatoms are drawing extensive attention in this field since they are expected to be one of the most relevant entry points of contaminants in marine food webs. Some of the most common pollutants in aquatic environments are the polycyclic aromatic hydrocarbons (PAHs), which display potential carcinogenic and mutagenic properties [73]. Especially in heavily urbanized or industrialized regions, the majority of PHAs arise from anthropogenic causes such as oil spillage, coal and wood burning, petrol and diesel oil combustion and other industrial processes [74]. DNA microarray based transciptomics were used to investigate the effect of sub-lethal concentrations of the PAH benzo(a)pyrene (BaP) on *T*. *pseudonana* [75]. The results revealed the regulation of genes related to oxidative stress responses, apoptosis, cell cycle and putative enzymes involved in BaP degradation as well as silicon and lipid metabolism. Genes involved in lipid biosynthesis and catabolism were up- and down-regulated, respectively, suggesting the activation of membrane repair mechanism to restore the membrane organization affected by incorporation of hydrophobic BaP [75]. It will be highly interesting in future work to chemically characterize the metabolite composition of diatoms treated by toxic contaminants in order to gain an integrated view of the global consequences of such contaminations.

## 4. Lipid Production and Potential Use of Diatom as a Source of Biofuel

### 4.1. Outlook of the Industrial Use of Microalgae

Large scale microalgal biomass and lipid production has been a topic of industrial interest for decades [76,77]. It is of particular importance due to the large number of metabolic products that can be obtained from microalgae. For example considerable research interest has been invested into biomass *per se*, polymers, food supplements, enzymes, toxins, coloring substances (pigments) and lipids [78,79]. Microalgae have also been harnessed for the treatment of wastewater and as a means to obtain “green energy” products [78,79].

**Figure 2:**
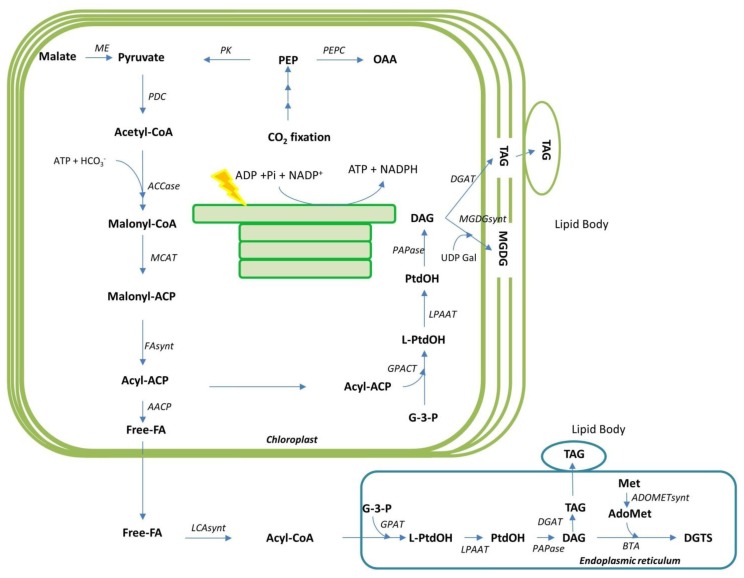
Simplified representation of the hypothesized triacylglycerol (TAG) biosynthesis and presumed subcellular compartmentalization in diatoms, which is inferred from the knowledge obtained from analyses in the green alga *Chlamydomonas reinhardtii*. Abbreviations: PEP, phosphoenolpyruvate; OAA, oxaloacetate; ACP, acyl carrier protein; CoA, coenzyme A; DAG, diacylglycerol; DGDG, digalactosyldiacylglycerol; Met, methionine; AdoMet, S-adenosylmethionine; DGTS, nonphosphorous betaine lipid diacylglyceryl-N,N,N-trimethylhomoserine; FA, fatty acid; G-3-P, glycerol-3-phosphate; L-PtdOH, Lyso-phosphatidic acid; MGDG, monogalactosyldiacylglycerol (a membrane lipid); PtdOH, phosphatidic acid; SQDG, sulfoquinovosyldiacylglycerol; UDP Gal, UDP-galactose; ME, malic enzyme; PK, pyruvate kinase; PEPC, phosphoenolpyruvate carboxylase; PDC, pyruvate dehydrogenase complex; ACCase, acetyl-CoA carboxylase; MCAT, malonyl-CoA:ACP transacylase; FAsynt, Type II fatty acid synthase components; AACP, Acyl-ACP thiolase; LCAsynt, Long-chain acyl-CoA synthetase; GPACT, Glycerol-3-phosphate:acyl-ACP acyltransferase; GPAT, acyl-CoA:glycerol-3-phosphate acyltransferase; LPAAT, lyso-phosphatidic acid acyl transferase; PAPase, phosphatidic acid phosphatase; BTA, betaine lipid synthase; DGAT, diacylglycerol acyl transferase; MGDGsynt, Monogalactosyldiacylglycerol synthase; ADOMETsynt, AdoMet synthetase. (Adapted and modified from [96] and [97]).

Due to increasing energy prices, which are at least in part driven by the limitation of the accessible fossil fuel reserves, there is a high demand for a renewable, carbon neutral, transport fuel, which is environmentally and commercially sustainable. Thus, the production of microalgae for biofuels emerges as a potential alternative to other sources since algae generally have high oil content and a rapid biomass production; they can grow in non-potable water and on non-arable land and therefore have small land area demand, in comparison with all other plant sources of biofuels [80,81]. The pathways for triacylglyceride (TAG) biosynthesis are well studied in *Chlamydomonas reinhardtii* (see Figure 2). Therefore, considerable research effort has been made to identify species of microalgae including diatoms, which are suitable for high lipid production [82,83]. These studies indicated that microalgae could accumulate between 20 and 50% of their dry mass as lipids, with this broad range being due to species and growth condition-dependent variability. Intriguingly, lipid content can even reach up to 90% of dry mass when cells are subject to physiologically stressful conditions, such as nutrient starvation or photo-oxidative stress [83,84,85]. These findings suggest that under cultivation conditions perceived as stressful by microalgae it may be possible to shift the metabolism from the use of photoassimilates for proliferation to their storage in energy storage compounds such as oil. Microalgae synthesize several types of lipids, however triacylglycerides (TAGs) are those of highest value for biofuel production since they can undergo *trans*esterification where TAGs react with methanol to produce biodiesel, fatty acid methyl esters [80]. Recent researches have demonstrated that fast growth and high lipid accumulation can be compatible by growing a productive culture under optimal growth condition to achieve high biomass and subsequently stressing the culture to induce lipid synthesis [86]. A similar strategy was recently postulated for biofuel production in plants following the realization that manipulation of the expression of members of the target of rapamycin (TOR) complex in Arabidopsis under the control of an inducible promoter was able to uncouple the enhanced lipid production it induces from the yield penalty observed following its constitutive manipulation [87]. Given that the TOR is well conserved in eukaryotes, this approach can also be applicable to algae. An alternative strategy to obtain high biomass and lipid production would be the selection or engineering of strains that concomitantly maintain high rates of lipid synthesis and growth [88]. This latter strategy is, however, likely to be a considerably higher risk since it requires simultaneous optimization for two independent objective functions and it is not understood yet whether these functions are mutually competitive or not.

### 4.2. Lipid Biosynthesis in Diatom Cells

#### 4.2.1. Pathways for Fatty Acid and Lipid Metabolism

Despite the sequencing of several microalgal genomes including a green alga, *Chlamydomonas* *reinhardtii*, a prasinophyte *Ostreococcus tauri*, a glaucophyte, *Cyanophora paradoxa* and two diatoms described above [13,14,89,90,91] (see list of the organisms in [92]), pathways related to algal lipid biosynthesis remain poorly characterized to date. In particular, little is known concerning the regulation of TAG formation at the molecular and cellular level in comparison with plants [83]. Based on genome sequencing efforts, the genes encoding a complete set of enzymes for several types of polyunsaturated fatty acid biosynthetic pathways were identified in *T. pseudonana* [13]. It includes several elongases and desaturases that modify relatively simple fatty acids. In addition, a sterol biosynthetic pathway that should produce cholesterol, cholestanol, and epibrassicasterol was predicted and a C-24(28) sterol reductase, that is probably involved in the synthesis of 24-methylene sterols was annotated [13]. Recently genes encoding acyl-CoA:diacylglycerol acyltransferase (DGAT), PtDGAT1 and PtDGAT2B, have been identified in *P. tricornutum* and characterized by functional complementation in yeast [93,94]. Thus these research efforts provided a molecular basis for TAG biosynthesis in diatoms although it is probably involved in other metabolic pathways. However in diatom cells the information of the TAG biosynthetic pathways is still limited so far and Chlamydomonas is known as a reference organism for algal TAG accumulation [95]. Therefore, we have described the hypothesized TAG biosynthetic pathways and presumed subcellular compartmentalization in diatoms in the Figure 2 according to what has been revealed for Chlamydomonas [96,97]. Recently a comprehensive survey of genes and emergent information related to algal lipid metabolism has been comprehensively reviewed [85]. On the basis of amino acid sequence homology and similarity of the biochemical characteristics of a number of enzymes related to lipid metabolism in algae and higher plants, it is believed that the basic pathways of fatty acid and TAG biosynthesis in algae are directly analogous to those which have been comprehensively described in higher plants (for a review see [83]). However, recent advances in molecular and biochemical analyses of microalgae also suggest several interesting differences in lipid metabolism between algal species and plants [97]. These differences are mainly in terms of the presence of betaine lipids and a more direct involvement of plastids in algae (Figure 2) [97]. It has also been proposed that, as a consequence, there are implications for the overall subcellular organization of glycerolipid metabolism [97].

For industrial purposes, interesting strains are those that produce 20% or more of oil relative to their mass. Diatoms can also store carbon as lipids [13,15] and have been reported to contain high amounts of lipids relative to other microalgae [98]. Therefore, they represent a highly promising source for biofuel production [82]. Also of high interest to the industry is the fact that diatom cells can produce a high proportion of highly unsaturated fatty acids [99], including nutritionally important polyunsaturated fatty acids, such as docosahexaenoic (22:6) and eicosapentaenoic (20:5) acids [100]. The complete biosynthesis machinery for polyunsaturated fatty acids and an additional sterol biosynthetic pathway were identified in the genome of *T*. *pseudonana* [13]. Moreover, both polyunsaturated fatty acids and other TAGs were identified following metabolic profiling of the model diatoms species *T. pseudonana* and *P. tricornutum* [100]. Recently, it was determined that when *P. tricornutum* was cultivated under sulfur, silicon, nitrogen and phosphorus starvation, nearly 100 molecular species of polyunsaturated TAGs could be identified [101]. In this study, the ratios of symmetrical to asymmetrical TAGs in the cells were affected by the nutrient stress, suggesting changes in the ratios of positional TAGs and also in the proportions of their chemical enantiomers. Thus, this study indicated that by affecting their cultivation conditions, it is possible to modify the structure of TAGs produced by diatoms [101].

#### 4.2.2. Influences of Nutrient Deprivation on Lipid Accumulation

As alluded to above and similar to other microalgae, the production of polyunsaturated fatty acids, as well as neutral lipids by diatoms cells is stimulated under stress conditions caused by nutrient deficiencies such as nitrogen, phosphorus and silicon, and the same seems to be true for the production of TAGs. Moreover, salinity, growth-medium pH, physical stimuli, temperature, light intensity and growth phase of the culture can modify fatty acid composition and TAGs contents [83]. The major molecular species of TAGs observed in *P. tricornutum* under carbon starvation conditions comprised of 46:1, 48:1, 48:2 and 48:3 species, with palmitic (16:0), palmitoleic (16:1) and myristic (14:0) acid constituents [100]. Additionally, this alga accumulates a certain percentage of long-chain polyunsaturated fatty acids in TAG molecular species with a higher degree of unsaturation, such as 20:4 n-6, 20:5 n-3, and 22:6 n-3 [100].

In *Chlamydomonas reinhardtii*, it has been shown that carbon is diverted to fatty acid biosynthesis under conditions of high C/N availability [102,103]. Additionally, it has been demonstrated that there is an increase in the expression of genes involved in lipid biosynthesis in *C. reinhardtii* when nitrogen is unavailable [104]. Interestingly, proteins related to the synthesis or degradation of these compounds were, however, not identified in *T. pseudonana* collected at early stages of nitrogen starvation [20]. The results from this study suggest that regulation of the abundance of glycolytic proteins appears to be differentially regulated in vascular plants and green algae although further research will be required in order to substantiate this theory. In addition, in a genome-wide transcriptome study of the *T. pseudonana,* no changes in the transcripts levels of genes related to lipid and fatty acid biosynthesis were found under nitrogen starvation conditions [105]. However, an increase in abundance of proteins related to the synthesis of acetyl-CoA, which is a precursor for fatty acid biosynthesis, was observed. These results thus suggest that an increase in fatty acid biosynthesis occurs only at later stages of the response to nitrogen deprivation [20] whilst an increase in the abundance of proteins related to glycolytic and TCA cycle enzymes was observed at earlier stages. This observation also indicates that the central carbon metabolism response of *T. pseudonana* to nitrogen starvation might differ considerably from that seen in plants and other eukaryotic photoautotrophic organisms studied to date. This finding is consistent with suggestions that diatoms can potentially use a multitude of strategies to deal with variations in cellular nitrogen levels [106], which includes differences in rates of protein turnover [107], and rearrangement in intracellular pools [108].

Given the nature of their cell walls, silicon is an important nutrient for diatoms, and it has also been demonstrated that there is an increase in lipid production of diatoms growing under silicon starvation [100,109,110]. However, detailed studies of the regulation of lipid metabolism in diatoms under silicon-limited conditions as a means of manipulating lipid metabolism remain scarce. Under silicon-depleted conditions of *Cyclotella cryptica* cell cultivation, higher levels of neutral lipids as well as higher proportions of saturated and mono-unsaturated fatty acids were obtained in comparison with silicon-replete cells [111]. In this study it was demonstrated that acetyl-CoA carboxylase (ACCase), which catalyzes the conversion of acetyl-CoA to malonyl-CoA, activity increased during silica limitation. Recently, potential target genes for miRNAs were identified in *P. tricornutum* cells growing under nitrogen and silicon deprivation, suggesting that miRNAs might play a role in fatty acid biosynthetic processes in this alga [112]. However, further research effort is required in order to both fully comprehend potential physiological roles of these miRNAs and to assess whether they would be a feasible target for attempts to influence lipid metabolism in diatoms.

Phosphorus starvation also leads to increased lipid content, mainly comprising increased TAG content, in marine algae, in both *P. tricornutum* and diatoms of the *Chaetoceros* sp. [113]. The physiological responses to phosphorus deficiency in diatom cells are, however, currently poorly understood. Interestingly, a recent study suggests that *T. pseudonana* and other phytoplanktons can conserve phosphorus under limiting conditions by replacing phosphorus containing lipids with non-phosphorus containing sulfolipids and betaine lipids containing nitrogen [114]. This ability to adjust cellular phosphorus levels is an important adaptation to phosphorus deficiency which allows cells to conserve phosphorus by both adjusting the phosphorus levels and by actively recycling lipid phosphorus [115]. Transcriptome and proteome analyses of *T. pseudonana* demonstrated the molecular responses in which phosphorus deficiency resulted in changes in cellular phosphorus allocation through polyphosphate production, enhanced phosphorus transport, a switch to utilization of dissolved organic phosphorus through increased production of metalloenzymes and a remodeling of the cell surface through production of sulfolipids [116].

Generally, lipid and fatty acid content and composition are also affected by variability during the growth cycle [83]. However it has been reported that at least in the model diatom species *P. tricornutum*, culture age has little influence on the total fatty acid content [117]. Interestingly, analysis of fatty acid composition in batch cultures of *P. tricornutum* and *Chaetoceros muelleri* suggested that increasing age leads to increases in the levels of some saturated and monounsaturated fatty acids, and concomitant decreases in the levels of polyunsaturated fatty acids [118]. When taken together the wholesale changes evoked by such stress responses suggest that in the long term such stress cultivation may not represent the best practice for production of algal biofuel. An alternative approach, which has been demonstrated in *C. reinhardtii* [102], is the removal of other competing pathways. In their proof of concept study Wang and co-workers demonstrated that by using the *C. reinhardtii* starchless mutant (*sta6*), deficient in the essential starch biosynthetic enzyme ADP-glucose pyrophosphorylase, they were able to harvest TAG yields in excess of those in nitrate starvation experiments. This study thus suggests that the reverse genetic approaches and the rapidly advancing field of synthetic biology may well represent the best routes for optimization of biofuel production in algae.

## 5. Conclusions

Carbon and energy metabolisms of diatom have drawn particular attention from researchers due to their evolutionary background and environmental importance. Genome sequences of two diatom species enable us to develop a blueprint of the metabolic networks which includes predicted pathways of the whole central carbon metabolism from inorganic carbon acquisition to respiration [15,16]. Additionally an expanding experimental toolbox including molecular genetic approaches has been applied to evaluate the functionality and regulation of the pathways. These analyses indeed highlighted the distinctive metabolic network of diatoms composed of enzymes homologous to other photosynthetic organisms as well as animals and bacteria. It is most likely that such an optimized metabolic network represents a great advantage to diatoms to be one of the most successful organisms in the contemporary ocean. Further application of integrative “omics” analysis is promising to elucidate the physiological functions of those peculiar pathways and the regulation of the carbon metabolism at the network level [53]. These approaches can also be applied to investigate the metabolic changes under various growth conditions. Given the abundance of diatoms, data provided by laboratory experiments should be crucial to simulate the effects of the changing environment on the global phytoplankton productivity.

It is clear that our knowledge concerning lipid metabolism at the molecular level in diatoms as well as in other microalgae is still largely incomplete. However, the recent genome sequencing and analysis reveal that genes encoding enzymes involved in lipid-biosynthesis pathways are present in various algal genomes and already indicate distinct aspects from what is known in plants and other model organisms. However, biochemical analyses to verify the function of each involved component are currently lacking. Furthermore, it is likely that transcriptome, proteome and metabolome analyses of different species of diatoms under environmental stress conditions will provide regulatory aspects of each pathway involved in lipid metabolism. Such studies will furthermore enable successful genetic manipulation and metabolic engineering of diatoms as well as other microalgae for oil production and productivity.
